# Game On, Science - How Video Game Technology May Help Biologists Tackle Visualization Challenges

**DOI:** 10.1371/journal.pone.0057990

**Published:** 2013-03-06

**Authors:** Zhihan Lv, Alex Tek, Franck Da Silva, Charly Empereur-mot, Matthieu Chavent, Marc Baaden

**Affiliations:** 1 Laboratoire de Biochimie Théorique, CNRS, UPR9080, Univ Paris Diderot Sorbonne Paris Cité, Paris, France; 2 Ocean University of China, QingDao, People’s Republic of China; 3 CEA, DAM, DIF, Arpajon, France; 4 Université Pierre et Marie Curie, UPMC-Sorbonne Universités, Paris, France; University of Edinburgh, United Kingdom

## Abstract

The video games industry develops ever more advanced technologies to improve rendering, image quality, ergonomics and user experience of their creations providing very simple to use tools to design new games. In the molecular sciences, only a small number of experts with specialized know-how are able to design interactive visualization applications, typically static computer programs that cannot easily be modified. Are there lessons to be learned from video games? Could their technology help us explore new molecular graphics ideas and render graphics developments accessible to non-specialists? This approach points to an extension of open computer programs, not only providing access to the source code, but also delivering an easily modifiable and extensible scientific research tool. In this work, we will explore these questions using the Unity3D game engine to develop and prototype a biological network and molecular visualization application for subsequent use in research or education. We have compared several routines to represent spheres and links between them, using either built-in Unity3D features or our own implementation. These developments resulted in a stand-alone viewer capable of displaying molecular structures, surfaces, animated electrostatic field lines and biological networks with powerful, artistic and illustrative rendering methods. We consider this work as a proof of principle demonstrating that the functionalities of classical viewers and more advanced novel features could be implemented in substantially less time and with less development effort. Our prototype is easily modifiable and extensible and may serve others as starting point and platform for their developments. A webserver example, standalone versions for MacOS X, Linux and Windows, source code, screen shots, videos and documentation are available at the address: http://unitymol.sourceforge.net/.

## Introduction

Biology currently undergoes a rapid expansion, calling for tools to visualize huge and complex systems, such as macromolecular structures, -omics networks or even organs and organisms [1]–[3]. It is a particular challenge for academic researchers to develop software solutions meeting these demands. At the same time, the video game and movie industries face similar needs in terms of complexity and efficiency to release products (i.e. respectively games and movies) for an increasingly wider audience. These industries benefit from more and more advanced tools enabling a quick development cycle, making the most of latest hardware and software. Can the scientific community use these tools to overcome complexity and efficiency issues related to software development by using solutions already provided by the entertainment industry? During recent years, this question was partially answered with respect to the movie industry as several research groups around the world have begun to use dedicated tools such as Maya (http://usa.autodesk.com/maya/), Cinema 4D (http://www.maxon.net/products/cinema-4d-studio.html) or Blender (http://www.blender.org/). Scientific projects associated to these programs include ePMV [4], Molecular Maya [5] and BioBlender [6]. Using such tools for animation is particularly beneficial for teaching as well as for communicating with a broad public, even leading to new discoveries [7] and valuable insight by combining experimental data and modelling [8]. Unlike the emerging spread of tools from the movie industry, the use of software to develop games remains largely unexplored in molecular graphics and modelling, despite its enormous potential.

For many years, game developers have gathered routines and frameworks that can be reused for a wide range of games. These code blocks have been aggregated to provide toolkits, called game engines, embedding all the components required to create a game in one package [9]. The core functionality typically includes a rendering engine for 2D or 3D graphics, a physics engine, sound, scripting, animation, artificial intelligence, networking, memory management, multi-threading, etc.… There are many game engines that are designed to generate executables for video game consoles, personal computers and even mobile devices: Unity3D (http://unity3d.com/), Unreal Engine (http://www.unrealengine.com/), CryEngine (http://mycryengine.com/), Blender Game Engine (http://www.blender.org/education-help/tutorials/game-engine/). These engines are free for non-commercial use and very appealing for developing scientific applications.

Here, we focus on molecular and network visualization using surfaces, spheres and links between them. With this test case we assess whether by using a game engine the functionality of classical viewers could be reproduced and extended in substantially less time and with less development effort than with common tools. Current needs include the ability to display hundreds of thousands of distinguishable elements in interactive time, using classical representation schemes. To provide a realistic scenario, inspired by current research, we have implemented our own visualization algorithm based on latest hardware capabilities such as programmable Graphics Processing Units (GPUs) [10]. The aim was to identify eventual restrictions imposed by the game engine programming environment. A user-friendly interface is required to facilitate navigation and interaction with virtual objects. In order to touch a wide audience, multiple platforms should be supported without the need to develop specific ports – ideally computers on Windows, MacOS and Linux operating systems, web pages, handheld devices such as smartphones and tablets. Most of the available game engines provide such features as well, but at different levels of development easiness.

We have chosen to test the Unity3D game engine (http://unity3d.com/) for its ability to deploy multi-platform applications with minimal programming effort. Furthermore, Unity3D provides an easy-to-use interface to develop 3D graphics applications using JavaScript, Boo – a python derived language - or C# code. Unity3D includes advanced functionalities to fully use the capacities of recent programmable graphics cards. This is possible employing optimized Cg functions [11], dedicated for Nvidia graphics cards, or GLSL (OpenGL Shading Language) code, a more universal language, which can be used on all recent graphics cards as well as on mobile devices [12]. The developer community is very active and helpful in providing example code and expert answers when technical problems are encountered.

In this article, we implement and compare several approaches for ball-and-stick representations using built-in Unity3D features. We also implemented the HyperBalls representation [13] and molecular surfaces. The advantages and limitations of each approach are discussed. Typical molecular properties such as the shape of molecules (surface) or their electrostatic potential can be visualized with original representation schemes. To demonstrate the flexibility of the development tool, we programmed a biological network viewer with interactive features. Using Lit Sphere shading [14], it is possible to achieve an artistic and illustrative rendering for any type of these representations. The outcome is a usable multi-platform stand-alone viewer that produces unique publication-quality figures and may serve as prototype for other developments.

## Methods

UnityMol can be deployed in two versions: a stand-alone application and a web applet running on top of the Unity3D web-plugin. We have tested several approaches to achieve the most efficient and appealing visualization of spheres, links and surfaces in order to balance graphical quality and display efficiency. We created a user interface to load and manipulate molecular structures, field lines, surface objects or biological network topologies.

### Accessing Data Files

For structural data we use the Protein Data Bank [15] format. Coordinate files can be imported from local storage or can be downloaded directly from the PDB server by entering their molecule ID. Due to security restrictions in the Unity3D web plugin, files have to be loaded through an intermediate server rather than from the local disk. After fetching files from the PDB or the server, they can be used by the web application. This restriction does not apply to the standalone viewer. PDB files are parsed to extract atom information such as atom type, residue name, residue id and coordinates. The molecule topology is built by using typical interatomic distances to detect bonds. Surfaces are generated either externally as meshes in the Wavefront OBJ format (these files contain vertex and face information used to reconstruct a 3D mesh), or internally using a density grid isosurface approach. Electrostatic potential grids computed by APBS [16] (or other tools) and stored in OpenDX format can be read for iso-surface visualization or be pre-treated using the BioBlender pipeline to generate Json files representing field lines. These files describe point coordinates for each field line to be animated. Network topologies are represented using extensible Graph Markup and Modelling Language (XGMML) format that can be obtained from Cytoscape (http://www.cs.rpi.edu/research/groups/pb/punin/public_html/XGMML/) [17]. XGMML files are parsed to extract id, name, position, size, colour and topology of nodes.

### Graphical Methods Used

We have tested graphical methods available in Unity3D as well as our HyperBalls representation based on ray casting [13]. The purpose of implementing HyperBalls was to evaluate how difficult it is to port previously developed code to Unity3D and whether this approach facilitates and speeds up the creation of a molecular graphics application. By implementing a molecular surface generation routine based on the classical marching cubes algorithm, we provide an additional proof of the generality of the game-engine approach for visualization applications.

#### Built-in graphical primitives

**Figure 1:**
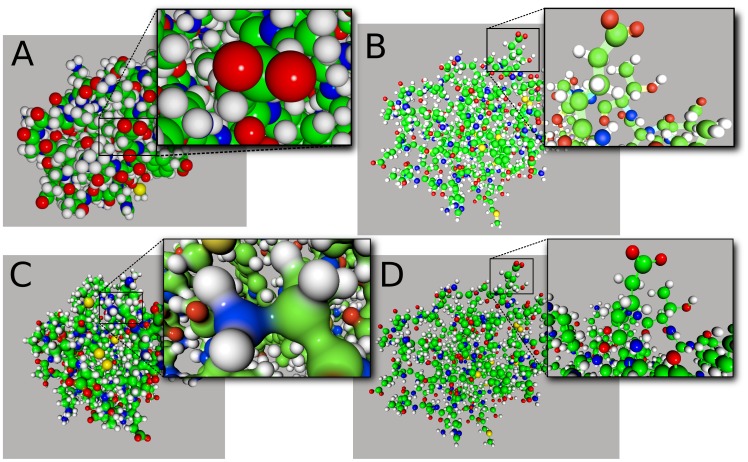
Different molecular representations implemented in UnityMol. A- Particle system depicting atom spheres. B- Unity3D triangulated spheres for atoms and lines for bonds. C- *HyperBalls* spheres and bonds. D- *HyperBalls* spheres for atoms and Unity3D cubes for bonds.

Unity3D provides an optimized set of graphical primitives for rendering. We use triangulated spheres, triangulated cubes and lines. Spheres and cubes display atoms (for molecular structures) or nodes (for networks); links between them are rendered using lines (see **Fig. 1** **-B,D**).

#### Optimized rendering with a point-sprite particle system

Tessellated graphical primitives require a large number of triangles to represent smooth spheres and are not appropriate to represent molecules within a game engine. Instead, a single square (composed of two triangles only) always oriented perpendicular to the screen plane can be used to represent a single atom. An image of the atom sphere called sprite is pasted onto the square. This point-sprite method is very efficient to display many similar images and can be extended to a so-called particle system (see **Fig. S1 in File S1**). In games, this visual method is commonly used to depict effects involving many small particles such as fire, smoke, clouds, snow, dust, etc. … For UnityMol, we have adapted Unity3D’s built-in particle system routine to paste spheres representing atoms on each square (see **Fig. 1-A**). In Unity3D, a single particle system is limited to 16 000 particles. For systems with more components, we implemented a mechanism to create and manage several particle systems transparently. This domain decomposition may lead to minor graphical artefacts as illustrated in **Fig. S2 in File S1**.

#### Using GPU shaders with the hyperBalls approach

We have developed a molecular representation called HyperBalls [13]. This representation takes advantage of the programmability of graphics cards using high quality ray casting with good display performance. The representation is composed of spheres depicting atoms linked by hyperboloid primitives rather than simple cylinders. Using such hyperboloids, the representation can be adapted to illustrate continuous bond evolution, passing from a one- to a two-sheeted hyperboloid (see **Fig. 1-C**).

The Unity3D framework enabled us to implement such a graphical method by integrating GPU code – so called shaders – to be run on the graphics card (as Cg or GLSL instructions). We implemented HyperBalls using built-in Cg functions. To create the representation, it is necessary to generate envelopes using cubes and parallelepipeds to define atom and bond primitives, respectively. Then, we represent spheres and hyperboloids within these envelopes using ray casting.

Starting from this previously published implementation, we were able to quickly add improvements to it, such as the lit spheres shading described below.

#### Visualization of electrostatic potential

Electrostatic potential visualization was implemented in UnityMol either as isosurfaces or as animated particles following field lines. The latter representation is similar to the rendering described in a recent publication by Andrei *et al* [18]. The lines are computed from particle advection inside a gradient vector field derived from an electrostatic potential grid computed by APBS [16]. Currently we use pre-computed field lines from BioBlender [18] stored in a text file. How to generate such files (using APBS or other software) is briefly described in **Text S1 in File S1**. We consider implementing the whole process in a future version of UnityMol, including interactive line selection filters. For now, only some representative lines are selected according to the electrostatic potential value on the molecule surface.

Once the line coordinates are loaded into memory, we reconstruct field lines using the line renderer of Unity3D. A dash-line effect depicting moving particles with a trail is achieved using a shader that animates a sinusoidal function along the field line according to a timer. The user can change the colour, the width, the length and the speed of the particles via the GUI (see discussion and examples of such lines in the results section).

#### Visualization of molecular surfaces

Molecular surfaces can be imported in Wavefront OBJ format from various molecular modelling programs such as MSMS [19], PyMol [20], Chimera [21], VMD [22] or PMV [23]. The 3D triangulated mesh is easily reconstructed using Unity3D routines and positioned to fit the atomic representation if one is loaded. Different methods can be used to render the surface using shader programs. By default, a simple lighting and a uniform colour are used.

Alternatively, UnityMol is able to generate a Gaussian molecular surface. An atomic density grid is computed by adding Gaussian distributions originating at the atom positions as discussed in [24]. This technique has subsequently been used in the Yasara software [25] and more recently also in the VMD program [26]. Then, the Marching Cubes algorithm [27] is applied to the density grid in order to generate an isosurface. The user can control the coarseness of the surface by changing the surface threshold. While the process is fairly fast for molecules up to 20 000 atoms, more optimizations are to follow for larger systems.

All types of surfaces can be cut according to fixed or moving planes. The interior of the molecule is filled by a uniform colour to enhance the user’s impression of cutting a solid object. The implementation uses a shader hiding the pixels of the mesh located above the plane defined by the user. The colour, orientation and position of the cut plane can be changed interactively (see examples in the results section).

For the moment, rendering of molecular surfaces is achieved by triangulation. The quality of such a surface is still limited because of memory restrictions. To improve this issue, it may be possible to build on recent works using the full capacity of modern graphical hardware to depict molecular surfaces combining a high quality of visualization and very good display performances [28]–[30]. It should be feasible to include such approaches in UnityMol in the near future, as the HyperBalls representation uses similar technology.

#### Lit sphere shading

On a virtual 3D object, the orientation of one pixel relative to the camera is determined by its normal. All the visible normals for one viewport can be retrieved on a theoretical half-sphere located at the centre of the screen. Thus a lighted sphere, or lit sphere, depicted in 2D contains all the information required to construct a lighting model. Sloan *et al.* [14] used this principle to light 3D meshes by mapping pixel normals with a 2D texture representing a sphere. In UnityMol, we implemented this approach for triangulated meshes and HyperBalls using shader programs to compute the normal for a pixel and retrieve the colour value at the corresponding coordinates on the texture. This value can be used as is or blended with other colours or textures to combine different rendering effects.

A few lit spheres are included as UnityMol program resources. The user can choose a texture for surfaces and HyperBalls representations using the GUI. In the standalone version, it is possible to import custom textures directly from the hard-drive.

### Hardware and Software Implementation

Development, benchmarking and testing were carried out on a Mac Pro computer with a quad-core Intel Xeon 3 GHz processor, 8 GB of memory, an NVIDIA GTX 285 graphics card, running 64bit Mac OS X 10.6. Results presented in this article are mostly based on the 3.5 version of Unity3D. All source code is implemented using C# and Cg languages built into Unity3D and is available on the sourceforge project website http://unitymol.sourceforge.net.

## Results

### Performance of Different Graphical Methods

In this section, we evaluate several Unity3D features on a simple test case and go on to determine the efficiency of the most promising features on a wider range of molecular structures. We then compare these features to our HyperBalls implementation.

#### Comparison for a simple test case: ferrocytochrome C

We used the molecule ferrocytochrome C [31], PDB ID: 1KX2, as a test case to compare the rendering approaches implemented in Unity3D. This protein is composed of 1 249 atoms and 1 113 bonds, a common size in the PDB.

**Figure 2:**
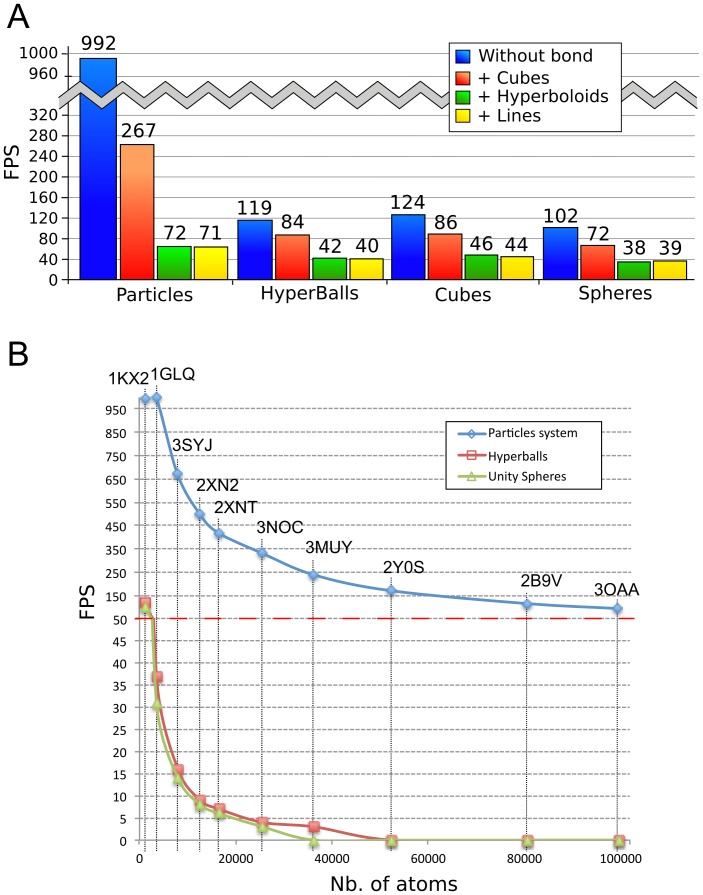
Benchmark of graphical methods. A – Rendering efficiency for ferrocytochrome C (PDB-id 1KX2) shown as atoms and bonds measured in Frames Per Second (FPS) for a 1344×1008 viewport. B - FPS values for molecules of increasing size rendered by a particle system, HyperBalls or Unity spheres. The red dashed line divides the graph in two parts: above this line the FPS step is 50, below this line the FPS step is 5.

We evaluated four methods to render atoms (see **Fig. 1**): a particle system, triangulated spheres, triangulated cubes and HyperBalls spheres. We also tested three methods to display bonds: cubes, lines and HyperBalls hyperboloids. The use of cubes is not of interest for representing atoms but it provides reference data for benchmarks allowing us to compare against the creation of simple graphical primitives with Unity3D. **Fig. 2-A** provides a benchmark showing that 3D point-sprites (particles) are the most efficient method to represent spheres in Unity3D. Triangulated cubes are eight times slower to render, closely followed by our implementation of spheres using HyperBalls and Unity’s built-in spheres. Rendering bonds in addition to atoms does not change this ranking, but we observe a marked decrease of display performance for the particle system, the display refresh rate being divided by more than a factor three. The impact is less important for the other methods, about a factor 1/3, using cubes for links. This value is divided by more than ten for the particle system when using hyperboloids or lines to render bonds. For the HyperBalls and Unity spheres representations, adding lines or hyperboloids divides the display rate by three. Concerning the particle system, these results can be explained by its high intrinsic efficiency to render spheres. The combination with other graphical methods puts a limit to this efficiency. In the other cases, the decrease in performance can be explained by the fact that the number of graphical primitives is nearly doubled when bonds are added.

For entirely fluid and interactive display manipulation, it is mandatory to achieve a rendering rate above 30 Frames Per Second (FPS). **Fig. 2-A** shows that the display performance with added bonds is acceptable for all primitives. Depending on the chosen method, the number of triangles and vertices for the graphical primitives may change considerably, directly affecting the display efficiency. The number of function calls to draw these elements is another important bottleneck with less calls leading to better efficiency (see **Table S1 in File S1**). Globally, these results depend on the size of the rendering window, and in particular concerning the HyperBalls representation, intrinsic graphics hardware performance is another key element that governs rendering performance.

#### Testing rendering efficiency limits and interactivity

We selected the particle system, Unity3D sphere and HyperBalls sphere methodologies to evaluate their respective efficiency limits using a set of molecules with sizes ranging from about 1 200 to 100 000 atoms (see **Table 1**). We focus on spherical atom representations of large macromolecular structures with the aim to render the maximum number of atoms in order to evaluate the brute performance of our own and of Unity3D built-in implementations. We assess the display rate for static molecules (see **Fig. 2-B**). As expected, the performance of the particle system exceeds the other two methodologies. Even for molecules as large as ATP synthase (PDB-id 3OAA), comprising 100 000 atoms, the frame rate remains high. The results for HyperBalls spheres are acceptable for molecules up to a few thousand atoms. Beyond 10 000 atoms, the number of frames per second (FPS) is near ten, the lowest rate at which rendering can be considered useable. We also analysed the impact of user interaction on display performance as described in supplementary material (see **Fig. S3 and Text S2 in File S1**). This lead us to improve the reactivity of UnityMol by implementing an on the fly optimization as described in the following paragraph.

**Table 1:** Set of molecules used to evaluate the rendering performance of UnityMol.

Molecule Name	PDB ID	Nb. of atoms	Reference
Ferrocytochrome C	1KX2	1 249	Bartalesi, et al. [31]
Glutathione S-transferase	1GLQ	3 516	Garcia-Saez, et al. [47]
Adhesin	3SYJ	7 968	Meng, et al. [48]
Alpha-galactosidase	2XN2	12 627	Fredslund, et al. [49]
Acetylcholine binding protein	2XNT	16 550	Akdemir, et al. [50]
DARPin bound to AcrB	3NOC	25 558	Monroe, et al. [51]
Beta-galactosidase	3MUY	36 360	Dugdale, et al. [52]
RNA polymerase	2Y0S	52 472	Wojtas, et al. [53]
Hydrolase	2B9V	80 710	Barends, et al. [54]
ATP synthase	3OAA	99 573	Cingolani and Duncan [55]

#### On the fly performance optimization using a level of details approach

To improve the overall reactivity of Unity3D, in particular for slower rendering modes such as HyperBalls spheres and bonds, we have implemented a so-called Level of Details (LoD) method. This functionality automatically switches from a specific representation that is assumed to render slowly to the fast particle system representation while the user is moving the molecule. This option is called “LoD Mode” in the UnityMol Graphical User Interface. LoD is commonly used in other generic data visualization packages such as Paraview (http://www.paraview.org/) to manipulate systems with many particles interactively.

### Molecular Features Visualization and Artistic Rendering

#### Electrostatic field lines visualization

**Figure 3:**
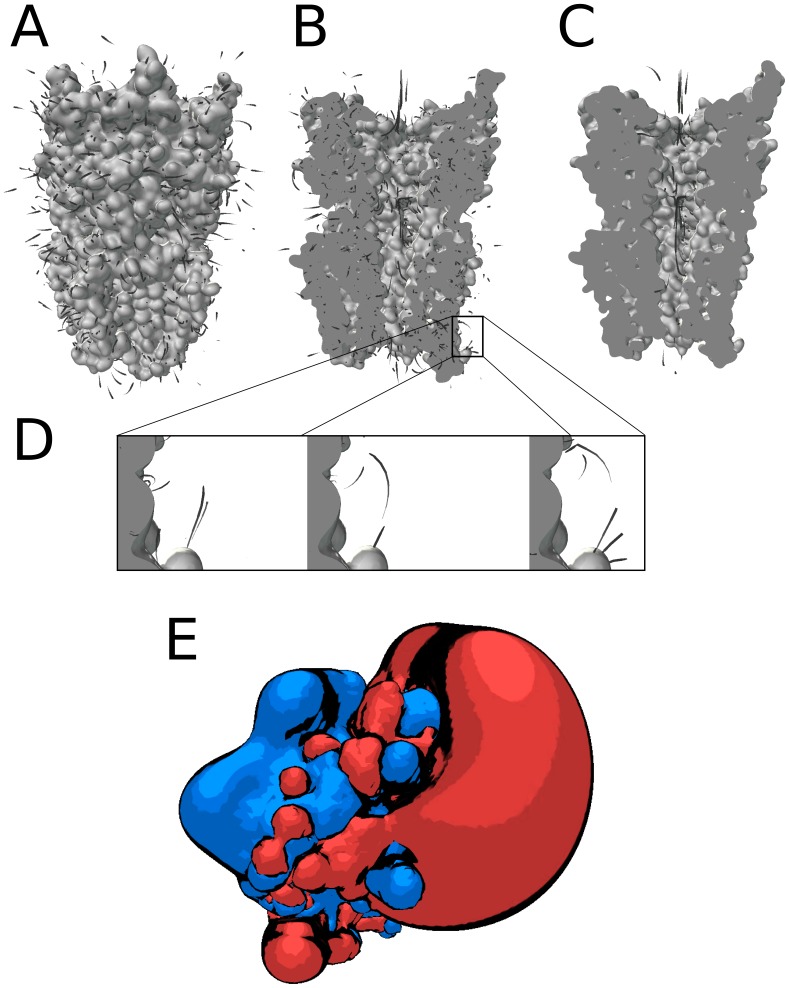
Visualization of animated electrostatic field lines and static iso-surfaces. A – Depiction of the electrostatic potential of the GLIC ion channel (PDB-id 3EAM) by animated trails moving along the field lines. B – Use of a cut plane to reveal the buried channel. C - The built-in Unity3D editor was used to remove some field lines and focus on the ones lining the channel pore. D – Time series showing 3 snapshots of a moving trail. E – Electrostatic potential of ferrocytochrome C shown as iso-surfaces (red : contoured at −2, blue : contoured at +2) with a toon shading to enhance the volume perception.

Recently, Andrei *et al.* have developed an animated representation to depict electrostatic field lines using the Blender program [18]. This visualization is particularly appealing and useful to represent the pathways that a charged molecule may follow guided by otherwise invisible electrostatic interactions. Unfortunately, the previous implementation is specific to the Blender package, which has a steep learning curve. Furthermore, it can only be run in an Internet browser window. We have reused this type of animation in the UnityMol viewer so that field lines can be visualized in the standalone version as well as in Internet browsers (see **Fig. 3**). Interactive features enable the user to change the graphical aspect of the field line animation.

In addition to dynamic field lines, classical static isosurface representations of electrostatic potentials can be rendered. The perception of the isosurface shapes may be enhanced using artistic rendering described below (see **Fig. 3E**).

#### Molecular surface cut planes

Molecular surface rendering is a fairly standard feature in molecular viewers. The addition of cut planes enables the user to easily visualize hidden elements in the interior of a molecule. This feature is particularly useful for proteins with internal cavities or channels (see **Fig. 3** **-B,C**) and when combined with the electrostatic field lines visualization. Very few molecular viewers implement this feature, which has been rendered particularly efficient and convenient to use in UnityMol.

#### Artistic rendering by lit sphere shading

**Figure 4:**
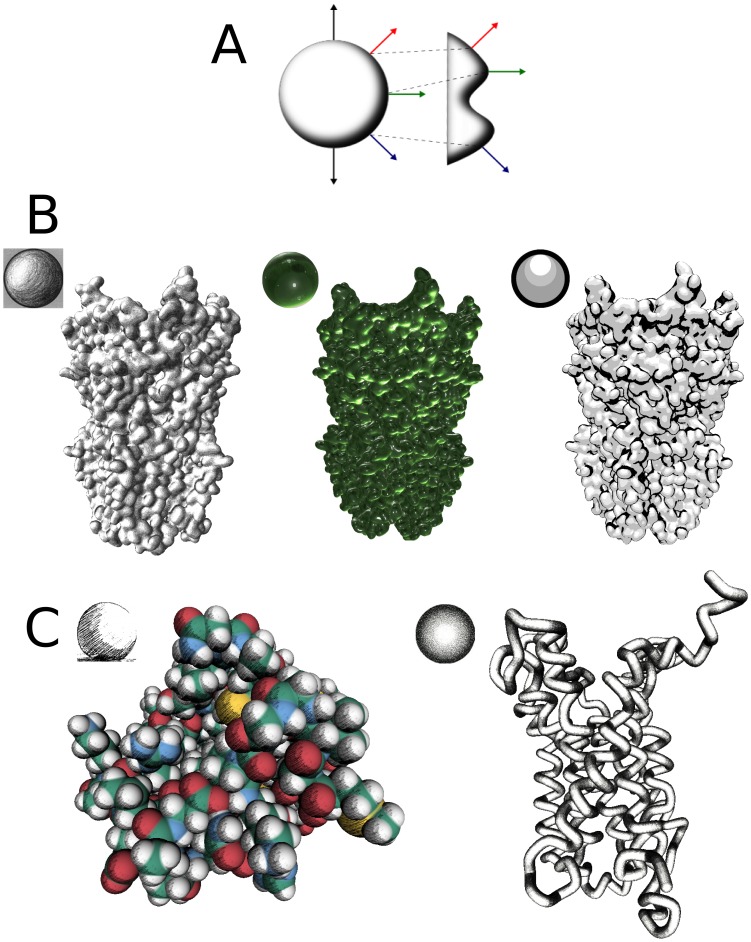
Lit spheres concept. A - All possible lighting and shading components of an arbitrary object (right) can be represented on a 2D image of a sphere (left). Colored arrows depict locations of equivalent surface orientation. B - Different lit-spheres applied to a molecular surface creating different effects : hand drawing (left), green glass (center), cartoon shading (right). C – Illustrative rendering using lit spheres and Hyperballs. Van der Waals (left) and backbone (right) representation of the BLT2 GPCR receptor with hand drawn lit spheres.

Lit sphere shading [14] is a rendering technique inspired from artistic methods to depict light and shadows on a three-dimensional object drawn on a two-dimensional surface. Based on the fact that a 2D depiction of a sphere encodes all the possible light values for a 3D object, we can use a 2D texture representing a sphere and its lighting, a so-called lit sphere, as a basis to light any 3D object. This process ensures a shading consistency and transfers all the artistic value put into the lit sphere onto the 3D object. For example, near ***photorealistic*** rendering is possible using spheres extracted from a photograph of a material such as metal, stone, glass etc… (see **Fig. 4**). As shown on **Fig. 4-C**, using a drawn sphere as reference texture produces an ***illustrative*** rendering close to visualizations generated by Weber [32] with a more demanding technique restricted to secondary structures. Bruckner et al [33] applied this technique to create style transfer functions that map different styles to different parts of a 3D object in a single rendering. This approach could be very useful to generate intuitive visualizations of molecular properties such as hydrophobic patches or to highlight specific binding sites within a molecule.

Lit sphere shading is a non-demanding method to render complex lighting effects in real time, using simple square textures. No performance loss has been observed while applying lit spheres onto a mesh or HyperBalls. As the light orientation is encoded in the lit sphere, the lighting effect is view-dependent which is desirable to achieve a consistent rendering when rotating the camera. An intrinsic inconvenience may be encountered when adding external light sources originating from a different direction than the one in the lit sphere.

### Game and Interaction Oriented Features

As a game engine, Unity3D provides pre-configured built-in features such as visual effects, a physics engine and access to peripherals (such as joypads). These functionalities can be reused for molecular visualization and interaction.

#### Visual effects

**Figure 5:**
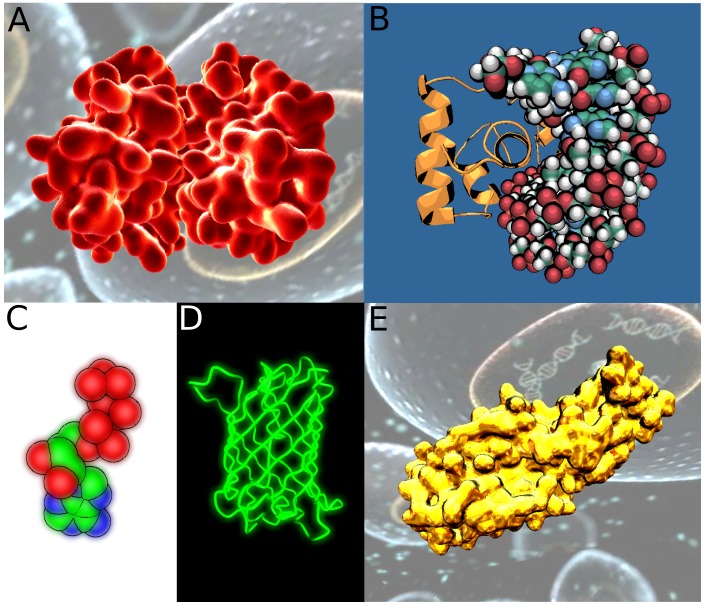
Several visual effects available in Unity. A – Human hemoglobin (PDB id: 1HHO) rendered with a stylized blood effect. B – Binding of a DNA molecule on the DNA-binding domain of Myb (PDB id: 1MSE). The secondary structure object was imported from PyMol. C - Contour outlining combined with glow. D – Green fluorescent protein shown as glowing carbon alpha spline (PDB id: 1KYS). E - Artistic molecule rendering : leucotoxin S component from *Staphylococcus Aureus* (PDB id: 1T5R) with a gold material. A,E – Background creation using a skybox.

There are two kinds of special effects in Unity: image based methods working in 2D screen space and methods working in 3D space. Using screen space effects, it is possible to deform an image, add black lines to outline contours or add glow and blur effects simulating persistence of vision (see **Fig. 5-C,D**). As an example of 3D space effects, we have added ambient occlusion to highlight protein shape (only available with triangulated primitives). Access to these advanced effects requires a Unity3D professional license for development.

A virtual environment may be created by adding a so-called Skybox surrounding the molecule and decorating it with landscape images (see **Fig. 5-A,E**).

#### Adding physical behaviour with a physics engine

In Unity3D, a physics engine is used to model physical and mechanical behaviour of rigid 3D objects such as collision, dynamics, elastic joints, etc. … To illustrate the potential of such an engine, we have implemented an interaction with the molecule based on a very simple spring network. The user can grab atoms to deform the molecule while the structure is kept coherent (“Interactive Mode” in the UnityMol GUI; see **Movie S1 described in File S1**). A visual feedback is provided by changing atom colours from white to black to represent the potential energy of each part of the elastic system.

#### Improving the user interaction experience through peripherals and GUI

Unity3D provides routines to use game peripherals such as joypads and joysticks. These game controllers can assist the user in navigating the scene as illustrated in **Movie S1 described in File S1**. UnityMol supports the joystick for control functions such as navigation *via* the integrated manipulator, selection of atoms and bond visualization modes, globally changing atomic radii and more. In the future, we plan to extend these functionalities using the Nintendo Wiimote, the Leap Motion and the Microsoft Kinect devices.

In Unity3D, it is straightforward to create a GUI without using a dedicated and potentially complex toolkit. We used built-in mouse picking functions to highlight selected atoms and access information (such as atom type, residue, etc.) or calculate distances between atoms. The UnityMol GUI, main program features and general usage of the software are described in **Fig. S4 in File S1**.

### Reusing Components for Speedy Development of a Systems Biology Application

**Figure 6:**
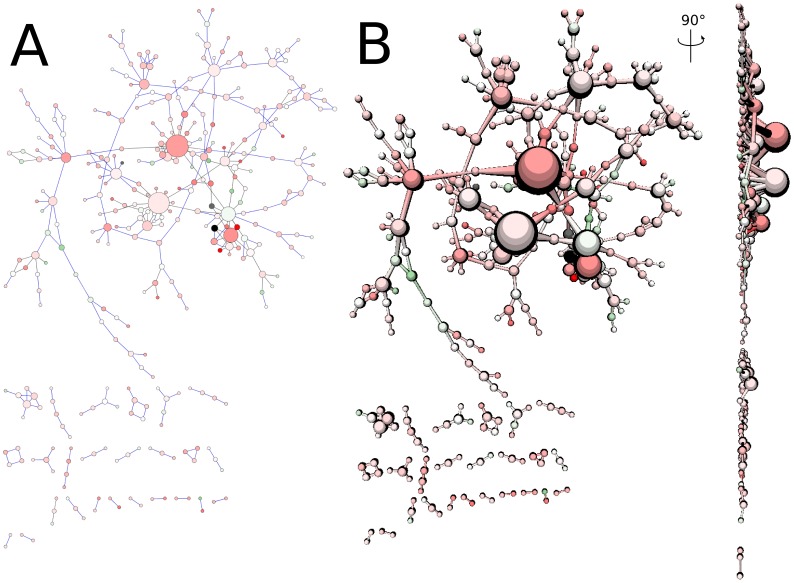
Biological network visualization. A – Original cytoscape visualization used as test case. B - UnityMol visualization with improved visibility by adding depth and contours using a lit sphere.

A particular benefit of a game engine is its modularity and the reusability of the generated routines and scripts. The use of an integrated intuitive editor facilitates the development of applications without writing numerous lines of code. It is possible to position 3D objects in the scene and apply scripts and graphical functions directly via the Unity editor. To illustrate this feature, we have adapted our molecular viewer to represent generic biological networks in 3D. To implement this network viewer (embedded in the demo), we have adapted the file parser to read XGMML (extensible Graph Markup and Modelling Language) format obtained as output of the CytoScape program [17]. The graphical primitives to represent atoms were used to depict network nodes while primitives displaying interatomic bonds are reused to model network edges. Colours and labels were adjusted to mimic conventions from Cytoscape (see **Fig. 6**). We vary the size of the nodes as a function of the radius provided by Cytoscape (depending on connectivity). We noticed that the default flat network view may create graphical artefacts as bigger nodes may hide adjacent smaller ones. To reduce this effect, we have implemented a function distributing nodes along the z-axis according to their radius (see **Fig. 6-B**). Hence, bigger nodes are positioned in the background or in the foreground (using the depth factor scroll bar in the GUI) to improve the visibility of the remaining nodes. We have reused the picking functionality to obtain specific network information when the user clicks on a node. The interactive mode presented previously can be used to manipulate the nodes and position them in a user-defined location (for convenient manipulation, choose a drag value of ∼5 and a spring value of ∼0).

## Discussion

### UnityMol a Graphical Application to Gather Molecular, Network and Electrical Field Lines Visualization

We have gathered recently developed graphical tools in an easy to use distributable software that provides a unique combination of functionalities absent from other publicly available viewers. We have implemented the recently developed HyperBalls representation [13]. In comparison to the original HyperBalls program, it is now possible to dynamically adapt the visual aspect to the user’s liking by continuously adjusting radius and shrink factor parameters via the graphical user interface. Furthermore, animation and artistic rendering enabled us to enhance classical molecular representations such as surfaces and electrostatic field lines. Additional visual effects allow the user to intensify a scene and create original images.

We compared our approach with common molecular viewers such as VMD and PyMol (see Table 2). These programs have better performances than our current version of UnityMol using GPU representations. Currently, the UnityMol Ball & Stick representation can interactively render molecules with few thousands of atoms. Hence, this particular representation will be more useful to create 3D graphical scenes for educational demonstration purposes as, in this case, the focus is often on molecules with a relatively small size. To visualize significantly larger systems it is recommended to use particle system spheres and switch on Hyperballs visualizations only when the user wants to obtain a very high quality figure. We have implemented the Level of Details method (see On the fly performance optimization part) precisely for this purpose. We are working to improve this part. The new version of the Unity3D game engine released a few weeks ago has already improved the display performances (see Table 2). We hope that future versions of Unity3D driven by the increasing need of video games for GPU power – to display new visual effects and accelerate interactivity – will help to further improve the visual performances of UnityMol. It should be emphasized that we present here a first version of our approach and we will continue work to develop more efficient algorithms – especially representations using the HyperBalls approach. We hope that our program may interest the community of computer scientists and game designers who may provide valuable help to overcome this type of limitations.

**Table 2:** Performance comparison between UnityMol using HyperBalls (with Unity 3D Engine Version 3.5 and 4), VMD and PyMol to display molecules with Ball & Stick representation on two different configurations.

MacOSX 10.6 - 2.93 Ghz GTX 285						Windows 7 - 3.0 Ghz GTX 480			
PdB id	Nb Atoms	UnityMol V3.5	UnityMol V4	VMD 1.9 glsl	Pymol 1.5	UnityMol V4	VMD 1.9 glsl	Pymol 1.5	
1KX2	1 249	44	52	198	135	35	272	330	
1GLQ	3 516	14	16	83	111	12	108	328	
3SYJ	7 968	7	8	37	109	5	51	325	
2XN2	12 627	4	4	24	70	3	32	321	
2XNT	16 550	3	3	18	68	3	24	314	
3NOC	25 558	3	3	11	51	3	16	299	
3MUY	36 360	3	3	8	44	3	11	278	
ZY0S	52 472	3	3	5	34	3	7	239	
2B9V	80 710	3	3	4	26	3	4	197	
3OAA	99 573	3	3	3	19	3	3	135	

In addition to molecules, we have implemented a 3D visualization of biological networks from Cytoscape [17] where the depth can be adapted to avoid overlap between different nodes. For the best of our knowledge, this is the first time that such a representation is used to display networks. All these depictions can be tuned using the simple graphical user interface to generate high-quality publication-ready images.

### An Easily Modifiable and Extensible Molecular Viewer with Editable Content

UnityMol is different from classical pre-compiled molecular viewer executables. In addition to the standalone and webserver versions, it can be run directly within the freely available Unity3D game engine. The game engine environment provides the user with full control over any detail of UnityMol. Because the Unity user interface is very intuitive, many simple things can be changed by the click of a button. It is straightforward to extend the application. If a GUI menu entry is missing, it can be added in minutes with just a few lines, most often by copying existing examples in the code. Moreover, when running UnityMol from within Unity3D, one can switch from game to editor mode and hand-edit the scene. Because the elements in the scene are Unity3D primitives, they can be modified on-screen by the user. For example, when visualizing field lines, the editor mode enables the user to select and delete field lines that are unwanted and hence fine-tune the scene (as in **Fig. 3-C**), despite the fact that we never explicitly implemented such a functionality (see **Fig. S5 in File S1**). This feature can be particularly useful to prototype new 3D molecular representations. Furthermore, instead of the need to recompile programs such as VMD or PyMol to see the results of modifications, using the Unity3D engine helps developers to modify, test and debug their code interactively using the editor window, on-the-fly compilation and the runtime debugger of MonoDevelop, the integrated development environment furnished with Unity3D. These features clearly help to simplify the visualization development due to a rapid and interactive trial-and-error test cycle. Furthermore, Unity3D provides simplified access to low-level graphics hardware functionalities that are usually not exposed in the APIs of existing programs such as VMD or PyMol.

Thus, such a wide range of control by the user could be seen as a new paradigm for scientific software development and presents a clear rupture with current approaches used for molecular visualization tools. At best, common packages provide scripting access to selected functionality to the user, otherwise programming know-how is required to delve directly into the application source code – if it is available. In the context of open computer programs [34], this approach provides users and developers not only with access to the source code but also with a very accessible interface (i.e. the editor) to examine the underlying science and implementation of the program.

### UnityMol as a Platform for Prototyping Scientific Tools

We have described how to use a game engine such as Unity3D to create a viewer for molecular structures as well as for biological networks. During the development of these tools, it became obvious that a game engine facilitates the development of scientific applications: the editor window provides an interface to position 3D objects and assist with the attribution of functionalities, e.g. implemented by a script, by dragging an icon onto the target object. Thus, the amount of lines of code to be written is rather limited and can in large parts be hidden for non-experts. Development is further facilitated by support from a very active Unity3D user community (see http://unity3d.com/support/community.html), which provides assistance to novice developers (this is also true for many other game engines). In particular the existing internet forums and answer sites are a good source of information with many pieces of code almost ready-to-use. For example, the original lit sphere shader was adapted from the official Unity3D forum. Several screen-space visual effects and the file browser are user contents as well. For non-expert programmers the availability of such resources speeds-up the development cycle and softens the learning curve of the programming environment. Many common visualization techniques are already implemented and provided with Unity3D such as triangulated spheres, lines, cubes and more, as well as a physics engine and built-in functions to manage different types of controllers. While the development tools are only available on Mac and Windows, stand-alone applications can be deployed for the Linux platform as well. There are some specific limitations in the free version of the Unity3D program (such as less visual effects). Buying a professional license provides access to special features or dedicated functions for systems such as game consoles or smartphones. Work is in progress in our group to explore these additional features and deploy UnityMol on new platforms such as mobile devices.

We only scratched the surface of many features that can be further extended to develop efficient programs. For example, the physics engine could be exploited to create spring networks, molecular dynamics and flexibility. Using this physics engine combined with joystick or Wiimote controllers, it would be straightforward to develop simple tools for interactive docking.

Our immediate plans are to extend UnityMol with practically important aspects for the biophysical community such as molecular dynamics trajectory loading and analysis. We started to experiment with the MDDriver library [35] to be able to connect to running or recorded MD simulations. Concerning analysis facilities (including atom selection and scripting) we intend to link UnityMol to existing frameworks such as for example MDAnalysis [36].

### And Designing Software for Edutainment

The video game industry is now booming and outperforms the movie industry with revenues reaching $18.6 billion in the U.S. in 2010. Interactivity is a key advantage that can be achieved with this medium. Direct interaction with the game itself as well as the creation of common virtual worlds where players can interact with each other contribute to this success. Such games can be a platform for education [37] as well as a place of study for academic fields such as social, behavioural and economic sciences [38]. Furthermore, scientific projects start to involve the general public in their investigations [39]. The structure of games is well adapted to involve a community to fulfil a particular task and may be used to resolve scientific issues. This approach has recently been employed to predict protein folding [40] and lead to impressive results [41]. There may be an enormous potential in such scientific “games”. Using adequate tools such as game engines may facilitate the creation of such games by scientists. Starting from the code base that we provide, it would be easy for a game designer working with Unity3D to create simple games for education or beyond. Furthermore, using dedicated game controllers such as the Wiimote and Kinect, will allow users to interact with molecules in a more natural manner. This may be a good solution to further implicate young people in classroom demo presentations or museum exhibitions. Interactivity is equally important for researchers to evaluate their hypotheses or develop new ones on live computer models by being able to “play” with them. Currently, interacting with models using tools dedicated for the movie industry is a difficult task given that making an animation may require several weeks, months or more [7].

### Concluding Remarks: Developing New Visual Paradigms to Deal with Big Data Challenges and Citizen Science

In the context of big data challenges, the development of citizen science – sometimes referred to as crowdsourcing - has been particularly welcomed and has proven its usefulness [39], [42], [43]. This approach is particularly promising in combining games with research applications in the biology and computer science fields and hence has already lead to major successes [41], [44], [45]. In this article we have shown that software usually dedicated to game development, so called game engines, can be used for 3D molecular visualization. This approach raises huge potentialities for quick developments which are particularly useful for the creation of small educational programs or simple serious games with a scientific purpose. The possibility to easily develop programs to automatically target major platforms (Linux, MacOS X and Windows) as well as media such as internet or video game consoles and nomad hardware (smartphones and tablets) reinforces the impact of this approach to touch a broad audience. The easiness of developing new visual methodologies – such as lit spheres presented in this paper – using the editor window can help developers designing new visual paradigms which are nowadays a priority to deal with challenges of data visualization [46]. These new developments can then be more easily included in well known molecular viewers such as VMD, PyMol, etc … Even if this first version is not as powerful as such molecular viewers, it opens a new way for developing visualizations and may pave the first step towards a convergence between game design and molecular visualization research.

## Supporting Information

File S1 **Describing 5 supporting figures, 3 supporting texts, 1 supporting table and 1 supporting movie.**
(PDF)
