# Supplementary material for: Game On, Science - How Video Game Technology May Help Biologists Tackle Visualization Challenges
Source: PLoS One. 2013 Mar 6;8(3):e57990. doi: 10.1371/journal.pone.0057990 (PMC3590297; doi:10.1371/journal.pone.0057990)
Supplement: File S1 — Describing 5 supporting figures, 3 supporting texts, 1 supporting table and 1 supporting movie. (PDF) [file pone.0057990.s001.pdf]

Game on, Science - how video game technology may help biologists tackle visualization challenges

Z. Lv, A. Tek, F. Da Silva, C. Empereur-mot, M. Chavent and M. Baaden

SUPPLEMENTARY MATERIAL

General remark: additional material as well as the UnityMol source code is accessible at http://unitymol.sourceforge.net.

CONTENTS

Supplementary Figure S1

Supplementary Figure S2

Supplementary Text S1

Supplementary Table S1

Supplementary Figure S3

Supplementary Text S2

Supplementary Movie S1

Supplementary Figure S4

Supplementary Figure S5

Supplementary Text S3

Supplementary Figure S1

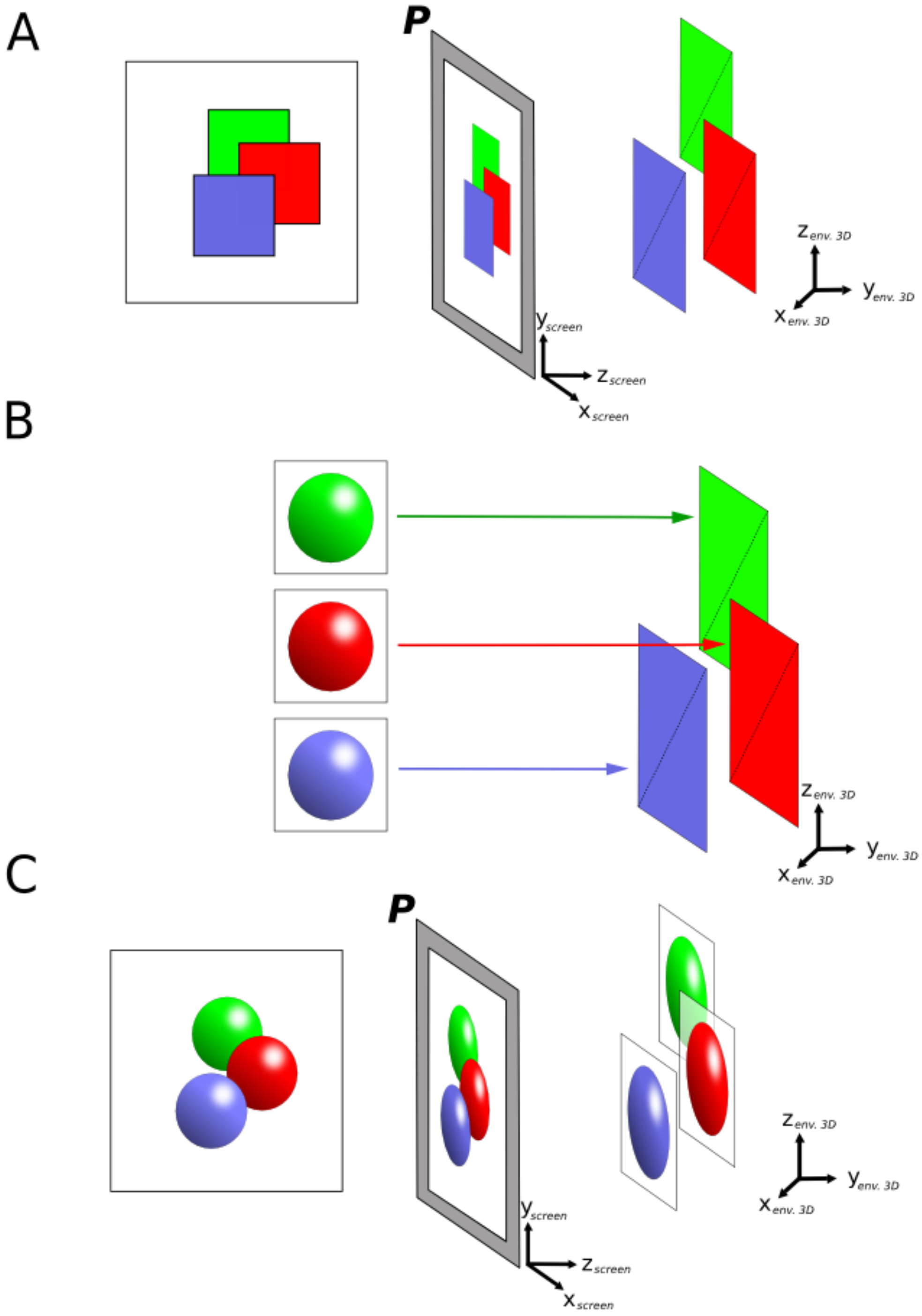

Point-sprite creation. A- Creation of squares oriented perpendicular to the screen. B – Pasting sphere images on squared planes. C – Visualization of the 3D point-sprites on the screen. For A and C, P represents the screen plane; on the left, resulting image on the screen and on the right, graphical primitives in the 3D environment. Hence, two environments are defined: a 3D-space environment and a screen-space environment.

Supplementary Figure S2

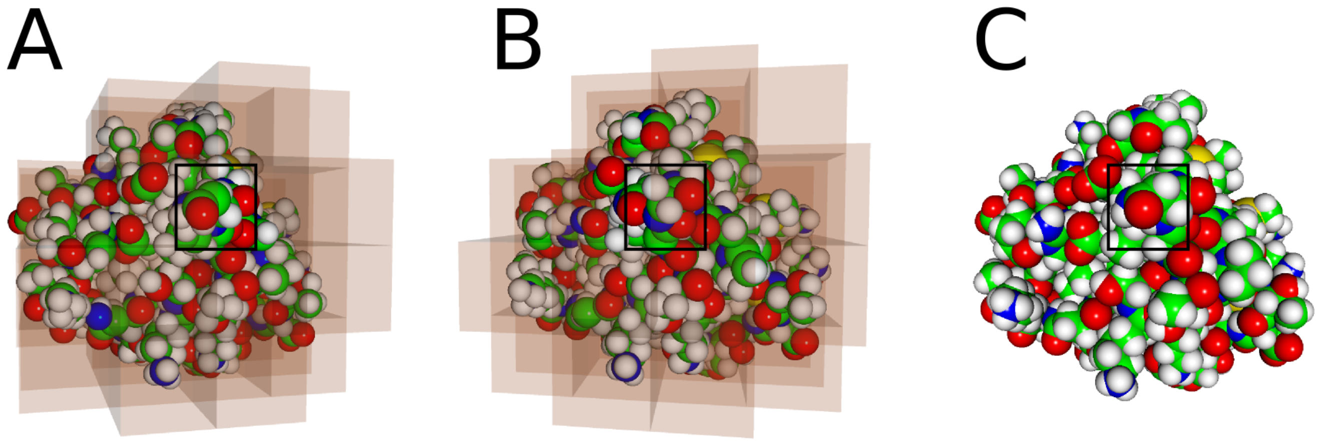

Particle system artefacts. Visualization of artefacts along domain frontiers (red planes) for the particle system representation with domain decomposition. A- Oxygen and carbon atoms are in the foreground (within the black rectangle). B- Rotating the molecule towards the left brings hydrogen and nitrogen atoms to the front. C- Correct intersections visualized with the HyperBalls representation.

Supplementary Text S1

How to generate animated potential fieldlines files (.apf)

From a pdb file, *yourfile.pdb*

Generate a potential grid for your molecule with the tool of your choice (e.g. : APBS; Delphi; UHBD etc.). The electrostatic potential grid data file must be in OpenDX format, which is the default output format for APBS. Conversion tools exist for other grid file formats. Name the potential grid file *yourfile.dx*

Generate a triangulated surface model of your molecule using Pymol, Chimera or MSMS and save it as a Wavefront OBJ file : *yourfile.obj*

To extract fieldlines, we use the pipeline proposed by the BioBlender software framework based on the work by M. Callieri *et al.*: Visualization methods for molecular studies on the web platform, The Web3D 2010 Conference, 22-24 July 2010, Los Angeles, California.

Download BioBlender (bioblender.eu) for your operating system.
Locate the program SCIVIS, for example in the 0.6 version of BioBlender :

> Linux :
> *BioBlenderv0.6_Linux/2.55/scripts/ui/BioBlender/bin/scivis/SCIVIS*
>
> MacOSX :
> *BioBlenderv0.6_MacOS/blender.app/Contents/MacOS/2.55/scripts/ui/BioBlender/bin/scivis/SCIVIS*
>
> Windows :
> *BioBlenderv0.6_Windows\Bin\2.55\scripts\ui\BioBlender\bin\scivis\SCIVIS.exe*

On the command line, run the following command to generate the type of field line data file used by UnityMol (.apf):

```
SCIVIS yourfile.obj yourfile.dx yourfile.apf 0.005 0.0 45 1 3.0
```

Place *yourfile.pdb*, *yourfile.obj*, *yourfile.dx* and *yourfile.apf* in the same directory.
Launch the UnityMol stand-alone program. Click Open. Browse through this directory and load *yourfile.pdb* (UnityMol automatically checks for and loads files with the .obj, .dx and .apf extensions).

Alternatively, it is also possible to use BioBlender entirely to generate such data by following the tutorial at

http://www.scivis.ifc.cnr.it/images/stories/3d_interactive/3D%20Interactive%20tutorial.pdf.

Supplementary Table S1

Suppl. Table S1: Number of primitives (triangles, vertices and draw calls) are shown for several rendering methods using the ferrocytochrome C molecule (PDB-id 1KX2).

Method Name	Primitive type	Draw Calls	Triangles	Vertices
Particle System	Atoms	1	2,5K	5K
Unity3D spheres	Atoms	1 249	949.2K	525.8K
Unity3D cubes	Atoms	1 249	15.0K	30.0k
HyperBalls spheres	Atoms	1 249	15.0K	30.0k
Unity3D cubes	Bonds	1 113	13.4K	26.7K
Unity3D lines	Bonds	1 113	2.2K	4.4K
HyperBalls hyperboloids	Bonds	1 113	13.4K	26.7K

Supplementary Figure S3

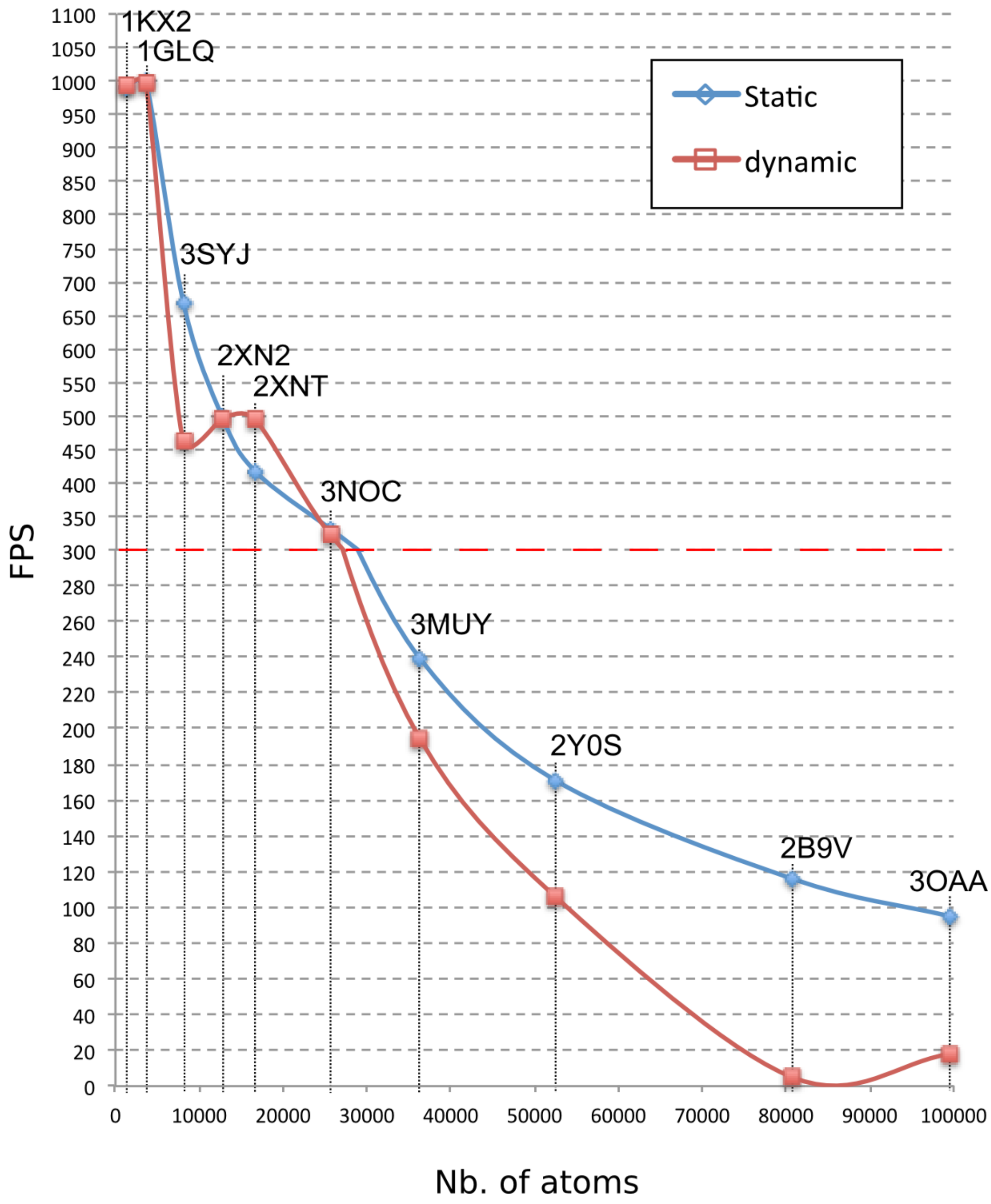

Static and dynamic benchmark. FPS values for static and dynamic rendering of the molecule dataset described in the main article by a particle system. The red dashed line divides the graph in two parts: above this line the FPS step is 50, below this line the FPS step is 20.

Supplementary Text S2

Display performance upon user interaction

We analysed the impact of user interaction on display performance by measuring FPS values for particle system spheres while the molecules are manipulated. To simulate user manipulation, we automatically rotate molecules at a speed rate of ~100 px/s mimicking user movements. Results are presented in **Suppl. Fig. S3**. The rate of movement has almost no effect on the performance of HyperBalls spheres, Unity3D cubes or spheres (data not shown), whereas it has a noticeable impact on the particle system. The impact is accentuated with the increase of molecule size, which can be explained by the fact that the depth sorting of all particles becomes increasingly time consuming. To compensate this scalability issue, we implemented domain decomposition by dividing the molecular structure into spatial blocks. Each block is depicted by an independent particle system. This implementation improves the efficiency for big molecules and enables the user to interactively manipulate molecules up to at least 50 000 atoms without a noticeable slowdown (see **Suppl. Fig. S3**). An inconvenience of the domain decomposition is the appearance of graphical artefacts at the frontiers between particle system domains (see **Suppl. Fig. S2)**. The calculation of depth used to determine the intersection between atoms depends on the centre of each domain. If the centre of one domain is closer to the screen plane than the centre of another domain, all atoms of this domain will be in the foreground with respect to all the atoms of the other domain. This behaviour may create artefacts at the frontier of these two domains. For large domains, the artefacts are mostly invisible as the frontiers are small compared to the whole molecule. For small molecules such as 1KX2 (presented in **Suppl. Fig. S2**) the domains are smaller and the artefacts are more apparent. For molecules with less than 4 000 atoms, the domain decomposition method is not useful and not desirable.

Thus, we used this domain decomposition in combination with the particle system to implement a level of detail approach where UnityMol switches from particles to HyperBalls as soon as movement stops. This is described in the main text.

Supplementary Movie S1

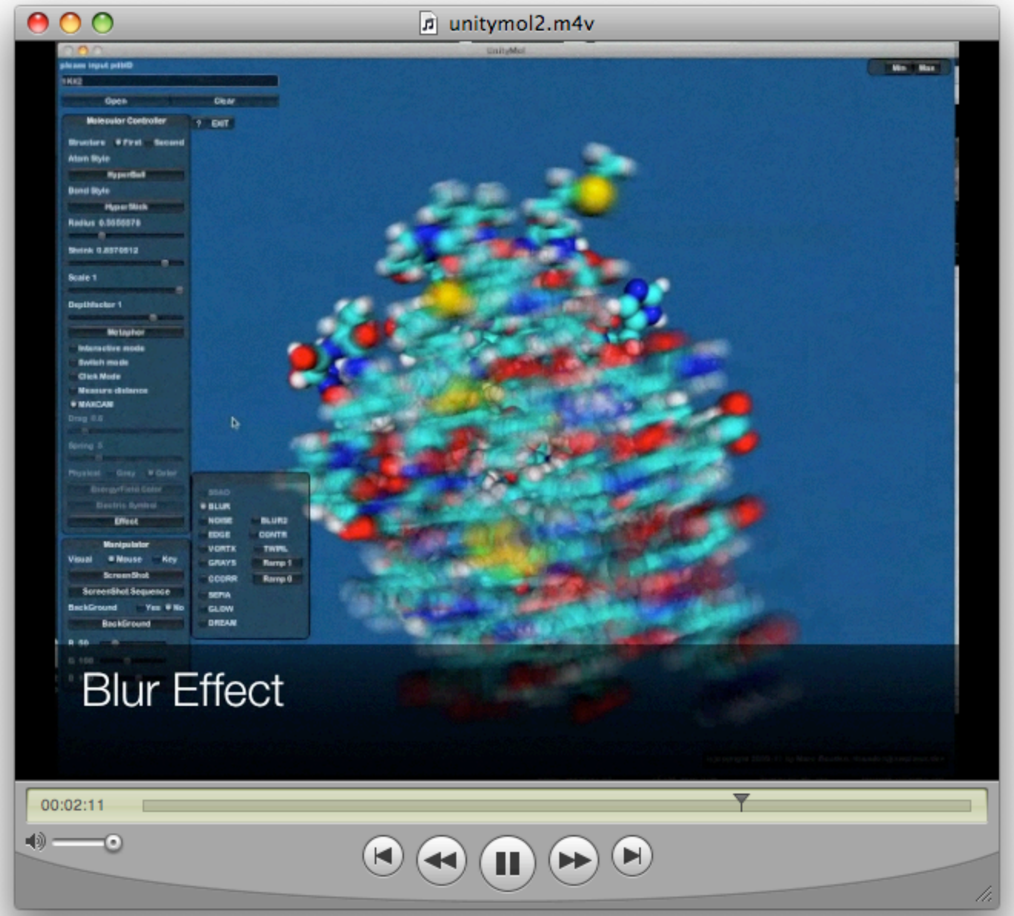

A demo movie illustrating many UnityMol features such as the interactive mode and the use of game peripherals for navigating the scene providing an overview of possible applications is available at http://www.baaden.ibpc.fr/pub/fvnano/umolmovie1.m4v or on the YouTube channel http://www.youtube.com/user/marcbaaden through the direct link http://www.youtube.com/watch?v=KA5XVllmobQ.

Supplementary Figure S4

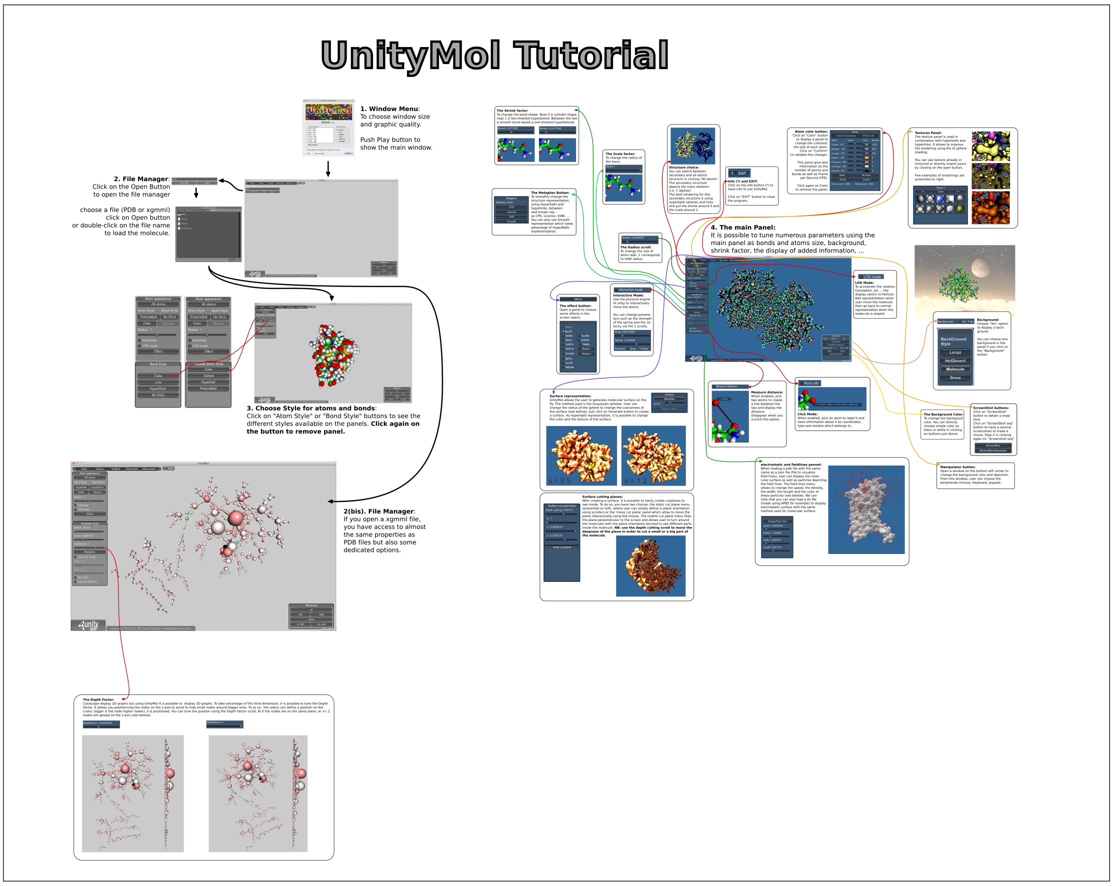

Tutorial to use UnityMol. Also available in full size at the following URL: http://www.baaden.ibpc.fr/pub/fvnano/umol_figs4.tiff.

Supplementary Figure S5

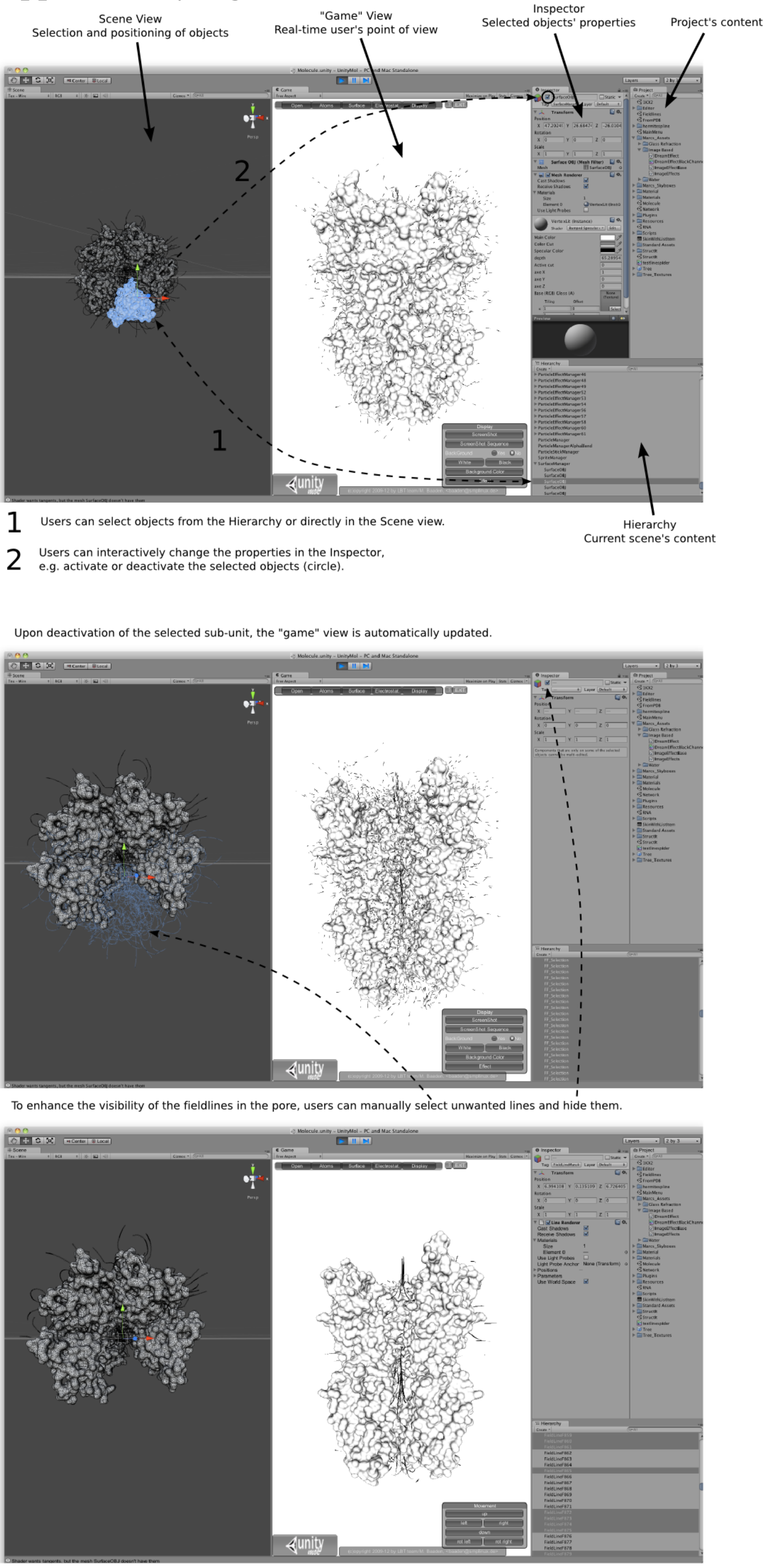

Editor mode. The figure illustrates how to edit a UnityMol scene in the Unity3D editor. It is thus possible to modify a running UnityMol application and finetune the visual scene, or modify and debug code interactively. In the example, several surfaces are loaded, one of which is deleted to reveal a channel and fieldlines driving ions through this channel. Subsequently, only the fieldlines of interest are kept, others deleted. Also available in full size at the following URL: http://www.baaden.ibpc.fr/pub/fvnano/umol_figs5.tiff.

<u>Supplementary Text S3</u>

Hidden graphical features with limitations

We have tested many more approaches than described in this paper: tubular bonds, multiple HyperBalls shaders on one object, mesh combinations of many objects, billboarded atoms etc. Yet, those implementations revealed defects and limitations of the game engine. By default they are hidden from the program menu. It is however possible to activate these additional choices on the left 'Atom style' and 'Bond style' menus by pressing the Delete key.
